# MiR-101 Induces Senescence and Prevents Apoptosis in the Background of DNA Damage in MCF7 Cells

**DOI:** 10.1371/journal.pone.0111177

**Published:** 2014-10-29

**Authors:** Siddharth Manvati, Kailash Chandra Mangalhara, P. Kalaiarasan, Niloo Srivastava, Bhupender Kumar, R. N. K. Bamezai

**Affiliations:** 1 School of Biotechnology, Shri Mata Vaishno Devi University, Katra, Kakrayal, Jammu & Kashmir, India; 2 National Centre of Applied Human Genetics, School of Life Sciences, Jawaharlal Nehru University, New Delhi, India; University of Barcelona, Spain

## Abstract

Moderately increased DNA damage due to the exogenous miR-101 (4 fold) over-expression in MCF7 cells was substantiated by an increase in the number of γ-H2AX foci, correlating with a simple-to-do Halo-assay. miR-101 induced mild/moderate DNA damage favoured senescence rather than apoptosis. An experimental support emanated from the induced mild/moderate DNA damage with 1 µM/5 µM etoposide in MCF7 cells, which resulted in an endogenous miR-101 over-expression (10/4 fold, respectively), followed by senescence. On the other hand, the severe DNA damage induced with 10 µM etoposide, resulted in a low (<1 fold) endogenous expression of miR-101 and an elevated percentage of apoptotic cells. Using bioinformatics tools along with *in-vitro* and *in-vivo* validations, miR-101 was found to target and downregulate the mRNA expression of UBE2N and SMARCA4, involved in DNA damage repair (DDR) pathways. Recovery of the expression of the two novel targets in anti-miR-101 transfection validated the results. We conclude that a threshold range of over-expressed miR-101, capable of inducing mild/moderate DNA damage, is sensed by cells to become senescent. The observation derives further support from *in-silico* protein-protein network analysis where the two novel targets showed their involvement in senescence pathway.

## Introduction

MicroRNAs (miRNAs) are small non-coding RNA molecules (20–25 nucleotides long) that regulate gene expression at post-transcriptional level [1], [2] by direct interaction at 5′ or 3′ UTR regions of target mRNA. The regulatory processes of miRNA, believed to involve around 60% of human genes [3], provide a new layer of regulation in gene expression. Understanding the role, therefore, of differential expression patterns of miRNA is of interest in cellular biology in general.

miR-101, one of the known microRNAs, was first reported to be down-regulated in prostate cancer and then in breast cancer cell lines, MCF7 and T47D, along with other miRNAs. The down-regulation of miR-101 was observed in several cancer types, such as Colorectal [4], Gastric, Lung [5], Bladder [6] and Breast Cancer [7]. Despite the knowledge about several of its targets, the direct mechanistic role of miR-101 in cancer and its specific interaction after the change in expression within a given pathwayhas remained elusive. Reports have also suggested an increase in expression of miR-101 under radiation induced DNA damage conditions [8]; and hypothesized its probable role in cellular senescence [9], not proven unequivocally. Here, we ascertain the role of this microRNA in inducing DNA damage which when mild/moderate guides the cells towards senescence instead of apoptosis. This biological role of miR-101 apparently is novel and of interest.

## Results

### miR-101 over-expression induces DNA Damage

**Figure 1:**
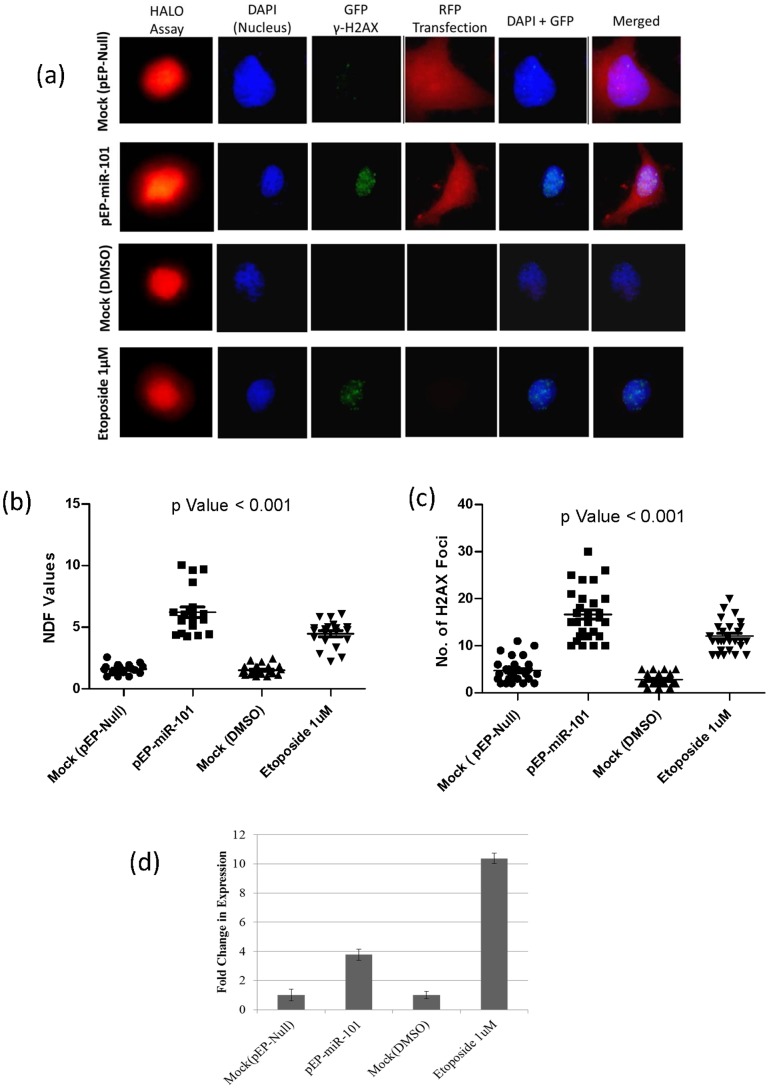
Visualization, quantification of DNA damage, and fold change in expression of miR-101 in MCF7 cells. (a) Fluorescence microscopy where six columns represent - Halo Assay used for calculating NDF values, DAPI staining for nucleus, γH2AX foci with GFP tagged secondary antibodies, RFP-tagged pEP-Vector, Merged-DAPI+GFP-tagged γH2AX and Merged-DAPI+ GFP-tagged γH2AX+ RFP-tagged pEP-Vector; (b) NDF values representing the extent of diffusion of damaged DNA in cells (Halo assay) [27]; (c) number of γH2AX foci; (d) fold change in expression of miR-101, in Controls: mock-pEP-Null-transfected, mock-DMSO treated, and Experimental conditions: pEP-miR-101 transfection and Etoposide 1 µM treatment.

A four-fold increase in miR-101 expression in transiently transfected MCF7 cells resulted in DNA damage, as assessed by the presence of an increased number of γH2AX foci and correlated with a simple-to-do Halo assay (Figure 1a), allowing semi-quantification through an increased nuclear diffusion factor (NDF) analysis. NDF values in miR-101 over-expressing cells showed a considerable increase (6.2±1.5) as compared to mock transfected (1.6±1.5) and etoposide (1 µM) treated cells, used as a positive control (4.4±1.5) (Figure 1b). The average number of γH2AX foci increased in miR-101 over-expressing cells (16±3) as compared to mock transfected (4±1) and the positive control (11±4) cells (Figure 1c), indicating the involvement of miR-101 in inducing DNA damage. The etoposide (1 µM) induced DNA damage in MCF-7 cells also resulted in an increased (10-Fold) endogenous expression of miR-101 (Figure 1d). The 4 to 10 fold exogenous or etoposide induced endogenous expression of miR-101, respectively, suggested the involvement of a threshold range of miR-101 in inducing mild/moderate DNA damage in the cell.

### miR-101 promotes senescence but not apoptosis

**Figure 2:**
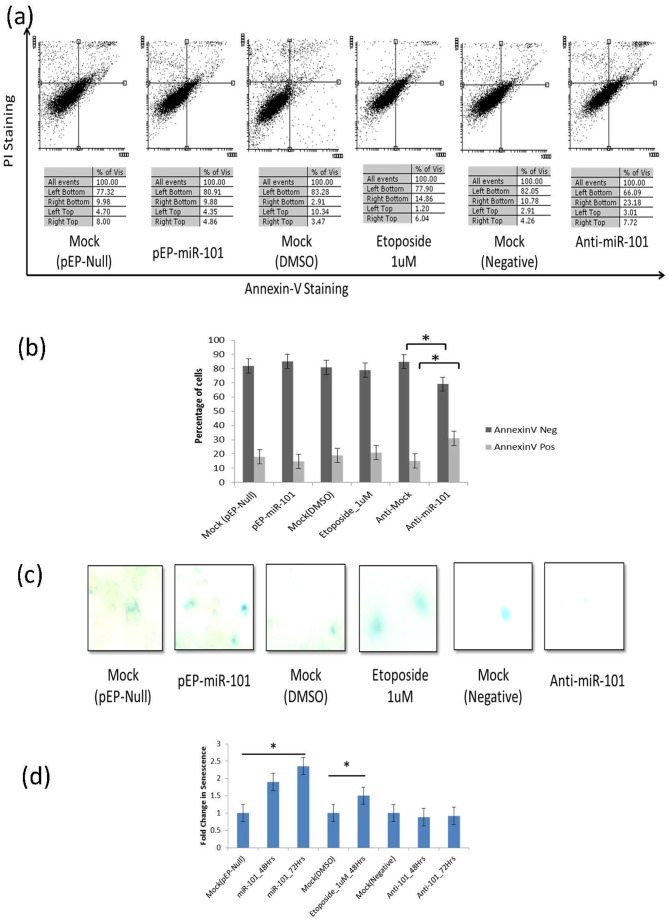
miR-101 induces senescence and not apoptosis in MCF7 cells. (a) Original Flow-cytometry dot-blot of PI Staining Vs Annexin-V Staining, (b) Percentage of Annexin-V positive (apoptotic) and negative cells, (c) β-gal positively stained (senescent) cells, (d) Fold change in number of senescent cells, under control (mock-pEP-Null-transfected, mock-DMSO treated & mock negative with scrambled primers) and experimental conditions (pEP-miR-101 transfection, Etoposide 1 µM treatment & anti-miR-101 transfection). Time dependent increase in senescence in miR-101 transfected cells is also shown (d).

Exogenously over-expression of miR-101 and etoposide (1 µM) induced endogenous expression of miR-101 in cells, resulting in mild/moderate DNA damage, was compared for the rate of cellular senescence or apoptosis. An absence of a significant difference in apoptotic cell population was noted between over-expressing miR-101 (15±2%), mock transfected control (17±2%), and etoposide treated (20±2%) cells. Whereas, inhibition of miR-101, using anti-miR oligonucleotides, increased the percentage of apoptotic cells significantly (30±2%) (p<0.05) (Figure 2 a & b).

**Figure 3:**
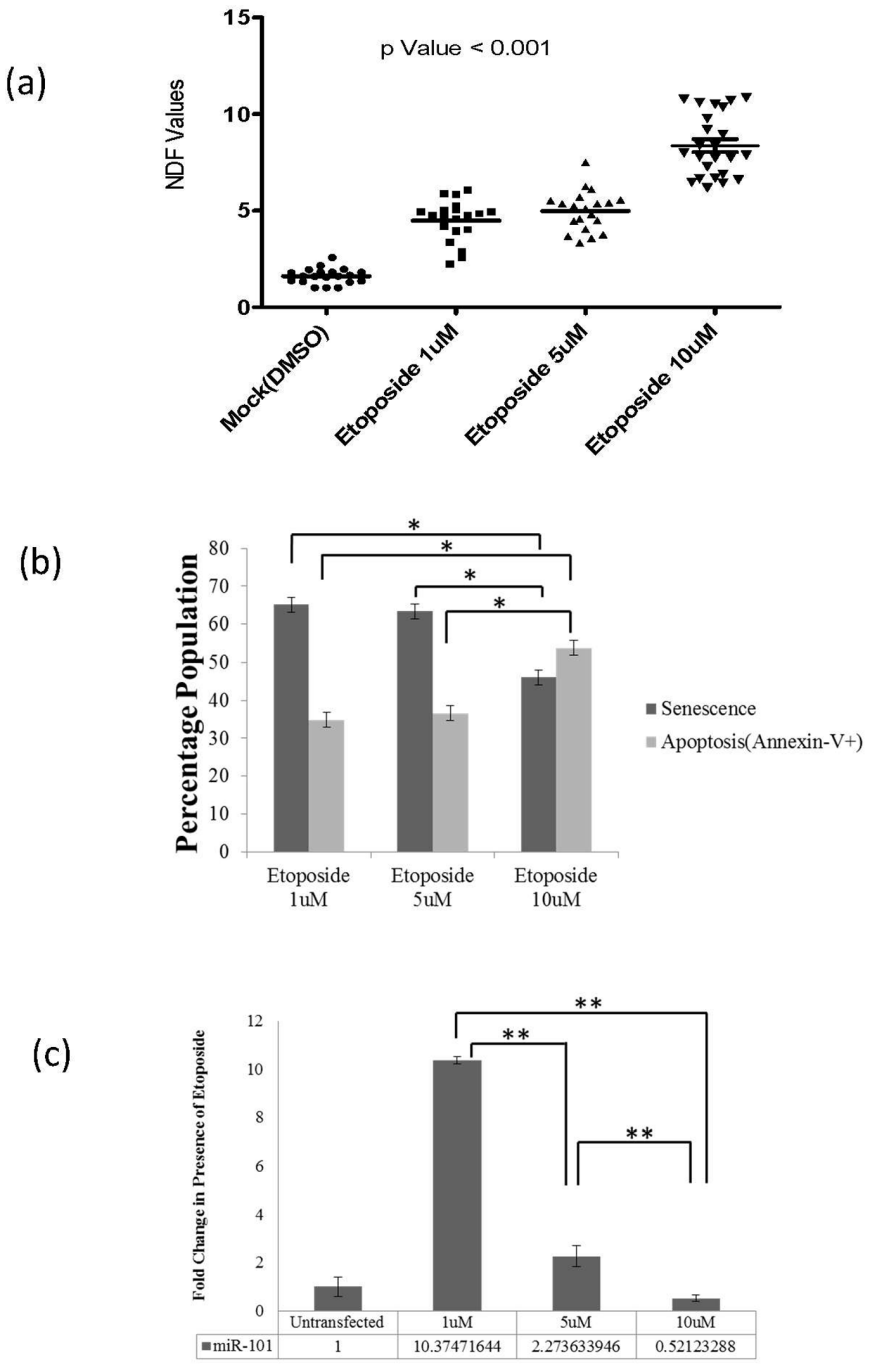
Halo assay performed in MCF7 cells. (a) NDF-nuclear diffusion factor-values showing the extent of DNA damage with increasing concentrations of etoposide (p Value <0.001), (b) Percentage of senescent cell population (using β-gal assay) and Annexin-V positive-apoptotic-cell population (through FACS analysis): showing decrease in senescence and increase in Apoptosis (*p Value <0.05), (c) miR-101 expression (using Real-Time PCR): showing decrease with increasing concentration of etoposide (1 µM, 5 µM and 10 µM) for 12 hrs (**p Value <0.005).

The number of senescent cells under similar conditions, however, showed a significant increase in miR-101 over-expressing (1.8-Fold ±0.25) also observed in 1 µM etoposide exposed (1.5-Fold ±0.25) MCF7 cells, when compared to mock transfected controls (p<0.05). Further, continuous (upto 72 hrs) over-expression of miR-101 depicted an enhanced induction of senescence (2.3-Fold ±0.25). miR-101 inhibition with anti-miR-101 oligonucleotides depicted a profile (0.9-Fold ±0.25) (Figure 2 c & d) which matched with the control cells. Further validation was carried out in experiments with well-established concentrations (1 µM, 5 µM and 10 µM) of etoposide inducing differential levels of DNA damage in MCF7 cells, which were assayed for (i) senescence, (ii) apoptosis and (iii) miR-101 expression. With an increasing DNA damage(average NDF values: 1 µM : 4, 5 µM : 5 and 10 µM : 8 in Halo assay) (Figure 3a), a concomitant decrease in the percentage of senescent cells(1 µM : 65±2%, 5 µM : 63±2% and 10 µM : 46±2%; p<0.05) and an increase in Annexin-V positive apoptotic cells (1 µM : 35±2%, 5 µM : 37±2% and 10 µM : 54±2%; p<0.05) (Figure 3b) was observed. Inhibition of miR-101 (with anti-miR-101) in 1 µM etoposide treated cells resulted in an increase in the percentage of annexin-V positive cells and a decrease in senescent cells (Figure S1 in File S1), supporting our observations.

The decrease in the endogenous expression of miR-101 experimentally with increasing doses of etoposide (1 µM: 10-Fold, 5 µM : 2-Fold and 10 µM : 0.5-Fold; p<0.005) (Figure 3c) correlated negatively with the induced DNA damage (Figure 3a) and the rate of apoptosis (Figure 3c). And, the results were positively correlatedwith increasing senescence (Figure 3c). Since, the generation of stable lines with miR-101 did not succeed; we assumed that a constitutive expression was not in favour of cell survival.

### miR-101 targets 3′UTR of UBE2N and SMARCA4

**Figure 4:**
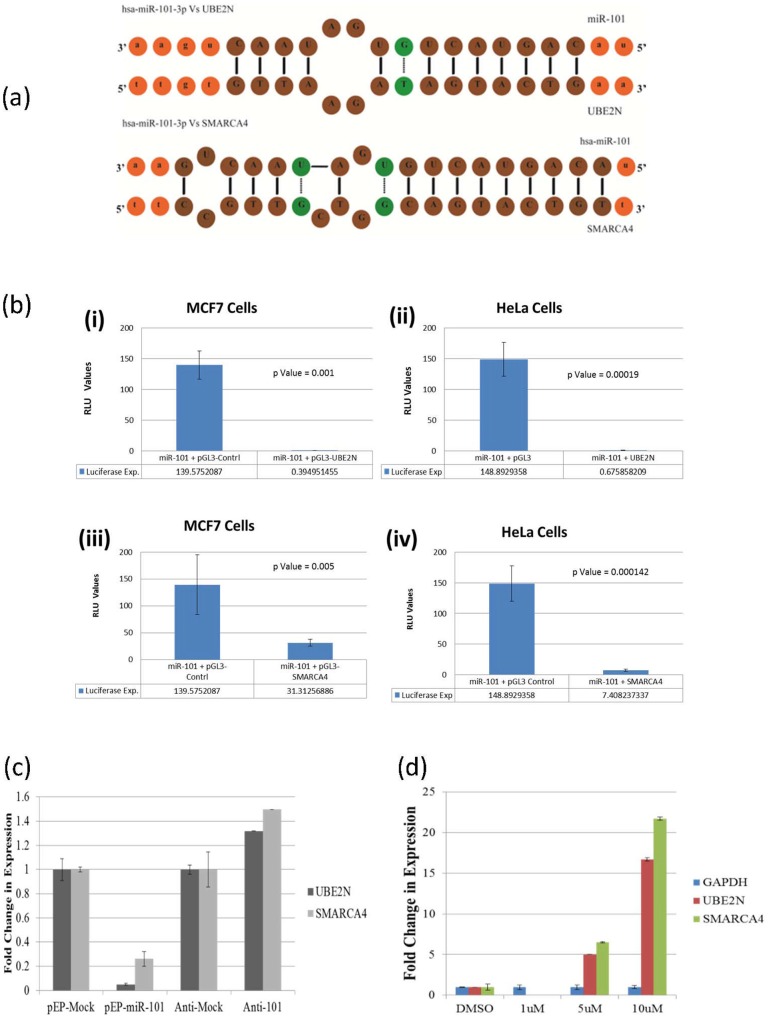
UBE2N and SMARCA4 as novel targets of miR-101. (a) Bio-informatics revealing the presence of miR-101 binding site at 3′UTR of UBE2N and SMARCA4, (b) activity of Luc gene showing a significant decrease in Luciferase activity when miR-101 was over-expressed in cells expressing Luc with 3′UTR of either (i & ii) UBE2N or (iii & iv) SMARCA4, in MCF7 and Hela cells. (c & d) Real-Time PCR showing a change in expression using Sybergreen and UBE2N and SMARCA4 specific primers, (c) under miR-101 and anti-miR-101 transfected conditions, showing decrease & increase, respectively, and (d) after 12 hrs exposure to 1uM, 5 uM and 10 uM etoposide, in MCF7 cells, confirming UBE2N and SMARCA4 as targets of miR-101, which is induced to 10 fold with 1 uM etoposide. At the higher concentrations of etoposide miR-101 expression is reduced leading to increased expression of UBE2N and SMARCA4, again confirming the relationship between miR-101 and the two novel targets.

In order to unravel the probable mechanism involved in miR-101 mediated DNA damage, gene specific targets of miR-101 were identified bio-informatically (Figure 4a) by three target prediction programs (described under Materials and Methods section). These targets were validated by *in-vitro* Dual-Luciferase Reporter Assays and the activity of luciferase measured from Luc gene in presence of 3′UTR binding site of UBE2N and SMARCA4, independently for the two genes, in presence of miR-101.The significantly decreased reporter gene activity in MCF7 cells (p = 0.001 & 0.005) and replicated in HeLa cells (p = 0.00019 & 0.000142), when compared to the control experiments where 3′UTR-binding-site was absent (Figure 4b), confirmed UBE2N and SMARCA4 as targets of miR-101. The validation of the two targets, UBE2N and SMARCA4, was established by measuring their endogenous cellular expression as well, which decreased significantly in over-expressing miR-101 cells (Figure 4c). The role of miR-101 in targeting these was further supported when etoposide (10 µM)-induced decrease in endogenous expression of miR-101 resulted in increased endogenous expression of the two cellular targets, UBE2N (1 µM : 0.003-Fold, 5 µM : 5-Fold and 10 µM : 16-Fold) and SMARCA4 (1 µM : 0.04-Fold, 5 µM : 6-Fold and 10 µM : 21-Fold) (Figure 4d). This concentration (10 µM) of etoposide not only induced low levels of miR-101 but also increased the rate of apoptosis, suggesting the least involvement of the miRNA in apoptotic processes.

**Figure 5:**
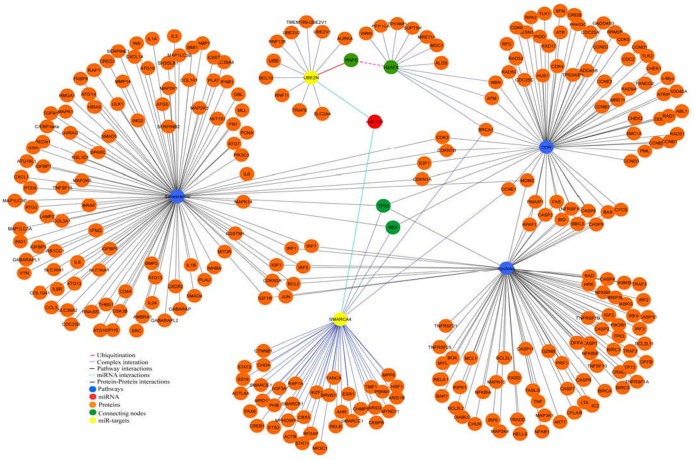
Protein-Protein network interaction of SMARCA4 and UBE2N as targets of miR-101 and as partners of proteins involved in DDR & senescence. Network explains the interaction of SMARCA4 and UBE2N with DDR pathway. SMARCA4 is shown to be involved in DDR and senescence pathways through RB1 and TP53. RNF8 forms a complex with H2AFX which is highlighted in pink colour edge and the ubiquitination of RNF8 by UBE2N is shown in red colour edge. All the nodes and edges of protein-protein interaction are in orange and violet colour respectively. The connecting key molecules in the network are highlighted as green. Pathway interaction nodes and edges are in blue and black colour respectively. The coloured node miR-101 which was predicted to target yellow-coloured-nodes, SMARCA4 and UBE2N, are highlighted by cyan colour edges.

*In-silico* protein-protein network analysis identified that the experimentally validated novel targets of miR-101, SMARCA4 and UBE2N, interact with proteins of DDR and senescence pathways via TP53 and RB1. One of the DDR pathway protein H2AFX, known to form complex with RNF8 [10], [11], was reported to be ubiquitinated by UBE2N [12] (Figure 5). *In-silico* network analysis also reflected a link between DDR and apoptosis related genes, as observed in experiments where, etoposide (10 µM) induced severe DNA damage resulted in low miR-101 expression and a high apoptotic rate. This process, *in-silico* and in experiments, did not involve the studied two targets of miR-101, suggesting the apoptotic induction to followed an independent path sever DNA damage in the cells.

## Discussion

A sequential role ofmiR-101 in inducing mild/moderate DNA damage followed by senescence in MCF7 cells was observed which has not been proposed earlier. Two independent experimental conditions, one of exogenous over-expression and second of endogenous induction with 1 µM etoposide of miR-101 in the cells, resulted in mild/moderate DNA damageand senescent cells. We propose that miR-101 is one amongst many of the candidates within the cellular milieu, which plays a significant role in initiating senescence and not apoptosis. The observations made by us lend support to the hypothesized role of miR-101 induced senescence [9], [13], [14] through DNA damage [15]. This event of senescence in a cell apparently happens under the condition of a mild/moderate DNA damage induced in a cell, which is shifted to apoptotic path in case the damage induced is severe.

The reversal of the phenomenon under anti-miR-101 experimental conditions, in presence of high dose (10 µM) of etoposide, where senescence decreased and apoptosis increased supported the role of miR-101 in senescence and not in apoptosis, the latter following an independent path. A decrease in the cellular mRNA expression of the two novel targets involved in DDR pathway, SMARCA4 and UBE2N, coincided with a 4–10 fold exogenous or endogenous expression of miR-101, resulting in mild/moderate DNA damage in cells which acquired senescence. Further increase in senescence in *in-vitro* experiments with longer exposure time of 72 hrs to exogenous over-expression of miR-101, supported the conclusions drawn. *In-silico* protein-protein network analysis, to look for the role of SMARCA4 and UBE2N and its interacting partners in DDR, senescence and apoptosis pathways revealed that P53 and Rb proteins interact with SMARCA4, the target gene of miR-101.P53 and Rb have been implicated in senescence[16]–[18] besides their role in cell death [19], [20] and survival [18], [21]. SMARCA4 (BRG1) has been implicated in cellular senescence by regulating pRb activity directlyor through upregulation of p21 [22]. Same is true for UBE2N, where MDC1 and H2AX complex, ubiqutinated by UBE2N [23]would fail to activate cell survival pathway, unable to form a complex with SMARCA4, RAP80, BRCA1, CCNDC98, to activate transcription of protein molecules involved in chromatin remodeling [19].

It would be interesting to understand in future if such senescent cells with mil/moderate DNA damage, could turn tumorigenic, since senescent cells have been reported to attain this characteristic [24]. Our study suggests, though the decisions towards neoplastic transformation or apoptosis may not necessarily be regulated by miR-101 alone; nevertheless its role in senescence after inducing a moderate DNA damage remains interesting.

## Materials and Methods

### miR-101 cloning in expression vector

miR-101 expressing vectors were generated using pre-miR amplicon of miR-101 from genomic DNA and pEP-miR vector (Cell Biolabs) as backbone. The pre-miR sequence was obtained using primer sets (Table S2 in File S1) and amplified from human genomic DNA by simple PCR reaction. The primers (IDT) were designed using online (Primer 3) and offline (Oligo) tools such that the 5′ ends of each forward and reverse primer contained Nhe-I and BamH1 restriction sites, respectively. These sites were also identified in pEP-miR vector for cloning purposes. After amplification the product was digested and cloned into pEP-miR vector, generating pEP-miR-101 recombinant clone.

### DNA damage induction and assays

Both miR-101 for exogenous (after transfection) and etoposide, one of the widely studied agents [25], [26], for endogenous induction of DNA Damage (as assessed in the present work), were used for experimentation. Cells were seeded and harvested in 6-well plate and supplemented with DMEM Medium with 10% FBS. After 24 hrs of seeding, the media was replaced with fresh DMEM with 10% FBS containing 1 µM/5 µM/10 µM concentrations of Etoposide, for 12 hrs. Etoposide powder was dissolved in DMSO according to manufacturer’s protocol.

#### Fast Halo Assay

After exogenous (miR-101) and endogenous (etoposide) induction of DNA damage, cells were suspended in ice-cold PBS containing 5 mmol/L EDTA and the suspension diluted with an equal volume of 2% low-melting agarose in PBS and immediately sandwiched between an agarose-coated slide and a coverslip. After complete gelling on ice, the coverslips were removed and the slides were immersed in 0.3 mol/L NaOH for 15 min at room temperature. Ethidium bromide (10 µg/mL) was directly added during the last 5 min of incubation [27]. The slides were then washed and de-stained for 5 min in distilled water. The ethidium bromide–labeled DNA images were acquired using a Fluorescnce microscopy (IX8I, Olympus) and processed with image analysis software (Image J). The extent of damage was quantified by calculating the nuclear diffusion factor (NDF), which represents the ratio between the total area of the halo plus nucleus and that of the nucleus.

#### γH2AX-Foci assay

In order to validate the extent of exogenously and endogenously induced DNA damage and correlate with the Halo-assay, the cells were fixed in 3% para-formaldehyde (PFA) (Sigma) in PBS (Sigma) for 15 min. Thereafter, cells were put in PBS (Sigma) and permeabilised on ice with0.2% Triton X-100 (Qualigen) in PBS (Sigma). Cells were washed three times in PBS (Sigma) containing 1% of Chicken Bovine Serum Albumine (BSA). Subsequently the staining was performed with a primary rabbit raised anti-γ-H2AX antibody (phosphorylated at Ser 139) (1∶300) (Invitrogen) for 1 h. After washing the cells three times in PBS (Sigma) containing 1% of Chicken BSA, the slides were incubated with a GFP tagged-polyclonal chicken anti-rabbit antibody (1∶1000) (Invitrogen) overnight. Afterwards, the slides were rinsed four times in PBS (Sigma). Finally, to counterstain the nucleus, 35 ml slow-fade mounting medium containing 2% 40,6-Diamidino-2-Phenylindole (DAPI) (Sigma) was dropped on the cells and slides were covered with a coverslip. Slides were scored for γH2AX-foci and preserved in a cool and dark place to allow the mounting medium to dry and to avoid fast fading of the fluorescent signal [28].

Images were viewed using Cell∧F Software and captured using digital camera of Olympus fluorescent microscope. All images were identically processed using auto features of the Cell∧F Software which has been used in automation of image analysis for Fluorescence microscopy. Only foci within the DAPI stained nucleus were counted. Deformed or fragmented nuclei, presumably corresponding to apoptotic cells, were excluded from the analysis. The vector used for transfection was RFP tagged and helped identify transfected cells. Cells were visualized by Olympus fluorescence microscope (IX8I, Olympus). A minimum of two images (∼15 to 50 cells per image) were taken randomly of each of four slides at 100X magnification.

### Apoptotic Assay

Apoptosis was measured by the flow-cytometer detection of phosphatidylserine externalization using APC Annexin V staining (BD Biosciences, MD). MCF-7 cells, after transfection with pre-miR-101, anti-miR-101 and control miRs, were harvested and processed for APC Annexin V staining as per the manufacturer’s protocol (BD Biosciences). Briefly, cells were washed twice with binding buffer (10 mmol/l (4-(2-hydroxyethyl)-1-piperazineethanesulfonic acid, 140 mmol/l NaCl and 5 mmol/l CaCl2, pH 7.4) and stained with APC-conjugated annexin V for 15 minutes at room temperature, followed by flow-cytometer analysis using the Becton Dickinson FACS Calibur (Franklin Lakes). The extent of apoptosis was quantified as the percentage of annexin V-positive cells [29].

### Senescence Assay

β-galactosidase assay allows the identification of senescent cells in mammalian culture cells [30]. Sub-confluent cells were washed twice with enough PBS to comfortably cover the cells (∼2 ml per 35 mm dish) for ∼30 s per wash. Enough fixation solution was added to submerge the cells (1–2 ml per 35 mm dish); and incubated for 5 min at room temperature. The fixation solution contained proportions of formaldehyde and glutaraldehyde, which are toxic and corrosive. The fixation solution was removed and fixed cells were washed twice with PBS. Staining solution (1–2 ml per 35 mm dish) was added and incubated overnight (12–16 h) at 37°C. After the incubation, the cells were washed for ∼30 s twice with PBS (2 ml per 35 mm dish), and once with methanol (1 ml per 35 mm dish) and air dried. The stained cells were viewed by bright field or phase contrast microscopy.

### Target Prediction & Validation

#### Bioinformatics prediction

The targets for miR-101 were predicted using three target prediction program (miRanda 3.3a [31], TargetScan 6.2 [32] and RNAhybrid 2.1 [33]). Fasta sequence of hsa-miR-101 was retrieved from miRBase. The binding site in the predicted targets for miR-101 was scanned using default parameters in all three software’s. 3′UTR sequences of SMARCA4 and UBE2N genes were retrieved from UCSC genome table browser. To identify the relationship between the DDR pathway with SMARCA4 and UBE2N, we constructed the network using cytoscape [34]. The DDR and senescence pathway proteins were retrieved from wiki pathway [35] and HPRD [36] database used to retrieve interacting protein of SMARCA4 and UBE2N.

#### Reporter Assay & target validation

The 3′UTR of novel targets, established bio-informatically, were cloned into the 3′UTR of pGL3-Control vector (Promega) at Xba-I site. The 3′UTR was amplified from human genomic DNA, using primer (IDT) sets (Table S1 in File S1). The amplicons were then cloned, generating pGL3-UBE2N & pGL3-SMARA4 vectors. All the clones were verified for containing the desired insert, by colony PCR, restriction digestion and DNA sequencing. Both pGL3 control and pEP-miR-101 (The miR-101 expressing vector) constructs were co-transfected in two different cell lines, MCF7 and HeLa. The transfection was performed using ESCORTS (Sigma-Aldrich) reagent and according to manufacturer’s protocol. The assay was performed after seeding in MCF7 and HeLa cells, in a 24-well plate, using Dual-Luciferase Assay Kit (Promega). After 48 hrs of transfection the cells were measured for Firefly and Renila luminescence, using luminometer. The ratio of Firefly and Renila reporter in presence of pEP-miR-101 was calculated and used in defining the change in expression of Firefly reporter in co-presence of predicted binding sites of specific genes.

#### RNA isolation and quantitative-PCR for miR-101 & target gene expression

RNA was isolated using TRIzol (Sigma) method and the quality and quantity verified by formaldehyde gel electrophoresis and Nanodrop (ND-1000, Nanodrop), respectively. cDNA preparation was carried out, using the High-Throughput cDNA Preparation kit (Part No-4368813, Life Technologies), following manufacturer’s protocol, using 5 ng of RNA for cDNA synthesis of microRNA. TaqManRNU44 and has-miR-101 assays were performed by real time PCR. SYBR green Real-Time PCR was performed using SYBR Green reaction mix and UBE2N & SMARCA4 specific primers, designed using both online and offline tools, i.e. primer 3 and Oligo, respectively. Target (miR-101) and reference (RNU44) genes were amplified using ABI PRISM 7000 sequence detection system (Applied Biosystems). For all Real-Time PCR reactions the threshold cycle (Ct) was obtained using SDS 1.1 RQ software (Applied Biosystems). The fold change in expression of the target microRNAs and the reference genes was calculated using Delta Delta Ct (ΔΔCt) method. RNU44 was selected as the endogenous control for microRNA expression analysis, whereas for gene expression analysis GAPDH was selected as the endogenous control.

### Statistical Analysis

The results obtained through Becton Dickinson FACS Calibur were analysed using Flowing software. The p values for miR expression were calculated, using Kruskel-Wallis test; and for luciferase assay by One-Way ANOVA.

## Supporting Information

File S1 **Figure S1,** (a) Percentage of annexin-v positive cells and (b) fold change in senescence in MCF7 cells, treated with 1 µM etopoiside independently and in combination with anti-miR-101. **Table S1,** Score of targets predicted by prediction tools. **Table S2,** List of primers used for cloning purpose.
(DOCX)
