# Supplementary material for: MiR-101 Induces Senescence and Prevents Apoptosis in the Background of DNA Damage in MCF7 Cells
Source: PLoS One. 2014 Oct 29;9(10):e111177. doi: 10.1371/journal.pone.0111177 (PMC4213038; doi:10.1371/journal.pone.0111177)
Supplement: File S1 — Figure S1, (a) Percentage of annexin-v positive cells and (b) fold change in senescence in MCF7 cells, treated with 1 µM etopoiside independently and in combination with anti-miR-101. Table S1, Score of targets predicted by prediction tools. Table S2, List of primers used for cloning purpose. (DOCX) [file pone.0111177.s001.docx]

**Table S1:** Score of targets predicted by prediction tools.

| **Prediction tools** | **SMARCA4** | **UBE2N** |
| --- | --- | --- |
| MiRanda-Score | 162 | 145 |
| MiRanda-Energy | -19.64 kCal/Mol | -14.98 kCal/Mol |
| TargetScan-Seed Match | 7mer-m8 | 7mer-1A |
| TargetScan-PCT | 0.46 | <0.1 |
| TargetScan-mfe | -24.2 kCal/Mol | -23.4 kCal/Mol |
| TargetScan-P-value | 1.00E+000 | 1.00E+000 |

**Table S2**: List of Primers used for cloning purpose

| **Gene Name** | **Primer Pairs** | **Tm** |
| --- | --- | --- |
| hsa-miR-101 | Forward:  5’-TCGAGGATCCGCTGGGCTCTGATCCTTCTT -3’  Reverse:  5’-TCGAGCTAGCCACCAACAACTACCCCATGTT -3’ | 60ºC |
| SMARCA4 | Forward:  5’-AATCTAGACAGCAGAGAAGCTGTAGGACTG-3’  Reverse:  5’-AATCTAGAGACAGGGACCTGGACATGAG-3’ | 62ºC |
| UBE2N | Forward:  5’-AATCTAGAAACAAACATCACAGCCCTCAC–3’  Reverse:  5’-AATCTAGACTCACAAATGCCTCAGCAAA–3’ | 62ºC |

**Figure S1**
